# Draft Genome Sequences of *Paenibacillus alvei* A6-6i and TS-15

**DOI:** 10.1128/genomeA.00673-13

**Published:** 2013-08-29

**Authors:** Yan Luo, Charles Wang, Sarah Allard, Errol Strain, Marc W. Allard, Eric W. Brown, Jie Zheng

**Affiliations:** Office of Regulatory Science, Center for Food Safety and Applied Nutrition, U.S. Food and Drug Administration, College Park, Maryland, USAa; Office of Analytics and Outreach, Center for Food Safety and Applied Nutrition, U.S. Food and Drug Administration, College Park, Maryland, USAb

## Abstract

Here, we report draft genomes of *Paenibacillus alvei* strains A6-6i and TS-15, which were isolated, respectively, from plant material and soil in the Virginia Eastern Shore (VES) tomato growing area. An array of genes related to antimicrobial biosynthetic pathways have been identified with whole-genome analyses of these strains.

## GENOME ANNOUNCEMENT

*Paenibacillus alvei* is a facultative spore-forming Gram-positive bacterium. It is ubiquitously present in the environment and has been isolated from a variety of sources, including cheese (1), fermented tomatoes (2), honey (3), rice plants (4), and soil (5). Many species in the genus *Paenibacillus* have been successfully used for agricultural, horticultural, industrial, and medical applications (6–8). *P. alvei* was also reported to produce peptide antibiotics that affect a wide spectrum of Gram-positive and Gram-negative bacteria (2).

Despite the increasing interest in *Paenibacillus* spp., genomic information for these bacteria is lacking. To date, only one whole-genome sequence has been reported for *P. alvei* in GenBank (9). More extensive genome sequencing might lead to the discovery of a rich source of genes with biotechnological potential. In the present report, we announce the availability of another two draft genomes of *P. alvei*. The two strains, *P. alvei* A6-6i and TS-15, were isolated from plant material and soil, respectively, in the Virginia Eastern Shore (VES) tomato growing area.

Genomic DNA was isolated from an overnight culture of each strain using a Qiagen DNeasy blood and tissue kit (Qiagen Inc., Valencia, CA). Genome sequencing was performed using 454 Titanium sequencing technology (Roche, Branford, CT), achieving >25× average genome coverage. A *de novo* assembly was created for each genome using the 454 Life Sciences Newbler software package v2.5.3 (Roche) and was annotated with the NCBI Prokaryotic Genomes Automatic Annotation Pipeline (http://www.ncbi.nlm.nih.gov/genome/annotation_prok/). An in-depth comparative genomic analysis of these data will be included in a future publication.

### Nucleotide sequence accession numbers.

The draft genome sequences of strains A6-6i and TS-15 are available in DDBJ/EMBL/GenBank under GenBank accession no. ATMS00000000 and ATMT00000000, respectively.
